# Preparation and Application of a Novel Slow-Releasing with Core-Shell Deicer in Asphalt Mixtures

**DOI:** 10.3390/polym14132615

**Published:** 2022-06-28

**Authors:** Yunxia Feng, Yuhong Luo, Junfeng Gao, Peng Guo, Yuntao Jiang, Fumao Liu

**Affiliations:** 1 School of Civil Engineering, Chongqing Jiaotong University, Chongqing 400074, China; cqjtufyx@163.com (Y.F.); lyh500232@outlook.com (Y.L.); 2 School of Materials Science and Engineering, Chongqing Jiaotong University, Chongqing 400074, China; jfgao@cqjtu.edu.cn; 3 China Gezhouba Group No.2 Engineering Co., Ltd., Chengdu 610091, China; nhgsazh@163.com (Y.J.); liufumao2022@163.com (F.L.)

**Keywords:** deicing asphalt mixture, slow-releasing deicer with core-shell, characterization, road performance, snow melting performance

## Abstract

The massive application of chloride salts has a direct effect on the corrosion of structures and vehicles and decreases durability as well as road pavement damage. A novel slow-release deicer with a core-shell structure was prepared to reduce the salts’ impacts, subsequently characterized by scanning electron microscopy (SEM) with energy dispersive spectroscopy (EDS), differential scanning calorimetry (DSC), and thermogravimetric analysis (TG). The conductivity evaluation, moisture absorption, and the snow or ice melting performance of the deicer were also tested. The core-shell deicer with different replacement rates was used to prepare the deicing asphalt mixture based on the equivalent volume replacement method. In this study, the high- and low-temperature performance, moisture damage resistance, and snow or ice melting capacity of mixtures were evaluated in the laboratory. The results show that the low-temperature and moisture stability performances decreased, and high-temperature performance improved, as the content of the core-shell deicer was increased. It is confirmed that the replacement rate of the deicer filler should be lower than 75% to meet the specification requirements. The prepared deicing asphalt mixture has good snow and ice melting performance and can reduce the bonding strength between ice and pavement surface. Durability and cost–benefit analysis are expected in further investigations.

## 1. Introduction

Asphalt mixture is extensively used for pavement construction all around the world [1]. Ice and snow in winter decrease the skid resistance between the pavement surface and tire [2], which greatly impact transportation safety, efficiency, traffic capacity, and people’s lives. With the requirements for travel, providing convenient and safe measures for removing ice and snow has become an urgent task for road maintenance in cold regions. Therefore, a variety of snow or ice removal technologies have developed around the whole world [3,4].

The commonly used methods of removing snow and ice on the road surface include labor and mechanical removal, elastic stress pavement, energy conversion type removal, and spraying chemical deicing agents [4]. Although the labor and mechanical removal are effective, they are also expensive and require special equipment. To a certain extent, using mechanical devices may reduce pavement performance and cannot remove the thin ice close to the road surface [2,5]. The U.S. Army Corps of Engineers Cold Regions Research Engineering Laboratory (CRREL) found that a rubber granules asphalt mixture can effectively remove the ice by changing the state between pavement and tires, and the effectivity is better with the increase of rubber granules content [6]. Unfortunately, the rubber granules asphalt mixtures were limited and rarely applied due to their durability. Furthermore, researchers had made a lot of efforts by heating the road surface to clear snow and ice, including cable heating, solar, or geothermal energy [7,8], microwave heating, and conductive asphalt mixture [9,10,11]. However, these methods have not been widely used due to their high cost and difficult maintenance.

Another universal approach is to spread chemical deicers, such as chlorine-based materials (sodium chloride, calcium chloride, magnesium chloride, and so on) and non-chlorine-based materials (e.g., urea, calcium magnesium acetate, sodium acetate) [12]. The chlorine-based deicers have various advantages, such as low cost, easy storage, and abundant resources, so they are widely used to remove ice and snow all over the world. Spreading chlorine-based deicers is not only expensive, but also time-consuming [13]. More importantly, chlorine-based deicers damage the pavement performance, corrode infrastructure, and pollute the surrounding soil and plants [14]. However, the non-chlorine-based materials are expensive and have low efficiency. Thus, the application of non-chlorine-based materials is mainly used for ice and snow removal on the airport runway [15,16].

In the 1980s, some European countries, such as Sweden and Germany, developed the chemical deicer whose main component is calcium chloride and freezing point is approximately −20 °C [17,18]. After that, based on the European deicer, Japan developed a new chemical deicer, Mafilon, whose main component is sodium chloride and freezing point is −10 °C [19]. With continuous in-depth research, researchers had further added deicers into the asphalt mixture. Since then, the self-ice-melting pavement was developed and was widely used [20,21]. In China, due to expensive prices and patent protection, some researchers tended to develop the deicers after the snow struck in 2008, such as sustained-release complex salt filler [22] and IceBane [23,24].

F. Giuliani [25] proposed that the chemical materials were released gradually and migrated from the inside to the outside of the asphalt film. Research [26,27,28] showed that the salt in deicers could be released from the asphalt mixture to inhibit freezing on the pavement surface. Liu et al. [29] showed that optimizing the mixing time could provide the best possible performance. If the mixing time of the anti-icing asphalt mixture was too long, the asphalt film on the particles would be too thick to release the anti-icing materials. Liu et al. [30] focused on the properties and salt-released characteristics under different moisture conditions of anti-freeze asphalt concrete (AFAC) containing anti-freeze filler. Xu et al. evaluated the effects and effectiveness of a sodium chloride-based anti-icing filler on andesite and limestone asphalt mixtures [31]. Xia et al. prepared the anti-freeze filler by carrier loading and AFAC. The results showed that when the filler replacement rate is less than 75%, AFAC has good water stability [32].

Laboratories and field investigations indicated that self-ice-melting asphalt mixtures had much better ice- and snow-melting ability than the traditional asphalt mixtures. Meanwhile, the literature also reported that the addition of deicer materials affected the asphalt mixture engineering performance, especially the low-temperature properties and moisture sensitivity [33,34].

Although the chloride-based deicers have obvious effectivity, the massive application of chloride salts has a direct effect on the corrosion of structures and vehicles and decreases durability as well as road pavement damage. In the previous studies, researchers focused mainly on the modification of the surface of deicer, including the hydrophobic modification. The effect of temperature and load on the material properties and salt release are not taken into account. Besides, Bayesian inversion is an efficient probabilistic model that can be used for parameter estimation, which has an important effect on the selection of parameters [35,36].

**Figure 1:**
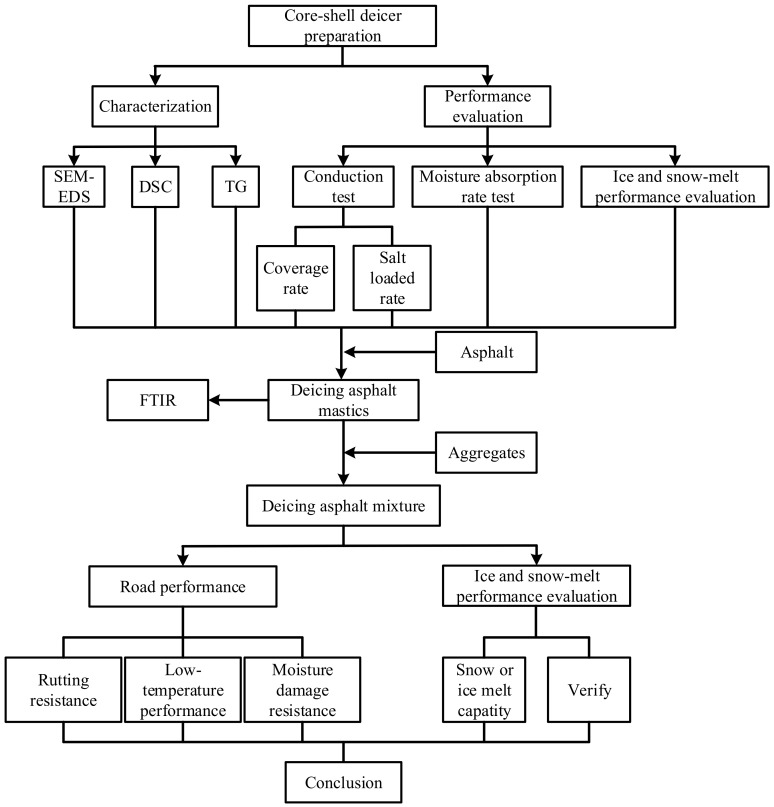
The research flowchart.

Based on existing deficiencies, a novel slow-release deicer with a core-shell structure was designed to achieve temperature and load control of the deicer. Firstly, according to the different solubility in a solvent, the salt in saturated solution is recrystallized in the pores of the carrier to form the slow-release inorganic salt. Next, considering the effects of the rains in summer, the polyacrylate shell was formed to protect the salt of the slow-release inorganic salt by in-situ polymerization. Based on the two steps, the slow-release deicer with a core-shell structure was prepared. Then, the slow-release deicer with a core-shell structure was researched and characterized. Finally, the slow-release deicer was added to the asphalt mixture to evaluate the engineering performance and snow-ice melting performance. The flowchart for the research is shown in Figure 1.

## 2. Materials and Methods

### 2.1. Materials

The raw materials were used for preparing the slow-release deicer with a core-shell structure including a carrier, deicing chemical, and other auxiliaries. The materials, such as diatomite, sodium chloride, OP-10 nonionic surfactant, nano-silica, ethanol, and distilled water, were used for preparing the slow-release inorganic salt. All chemical products were supplied by Shanghai Aladdin Biochemical Technology Co., Ltd., Shanghai, China. The deicing chemical is a chlorine salt, such as sodium chloride (NaCl), magnesium chloride (MgCl_2_), and calcium chloride (CaCl_2_). According to the eutectic and effective temperature shown in Table 1, sodium chloride was chosen as a deicing chemical.

The others, such as acrylic acid, methyl acrylate, butyl acrylate, isopropanol, silane coupling agent, azobisisobutyronitrile (AIBN), and triethylamine, were used for preparing the core-shell structure provided by Shanghai Aladdin Biochemical Technology Co., Ltd. Styrene was provided by Shanghai Macklin Biochemical Co., Ltd., Shanghai, China. Distilled water was from the laboratory. All reagents are analytically pure and used straightly.

The basic properties of SBS modified asphalt and limestone aggregates are shown in Table 2 and Table 3, correspondingly. The asphalt and aggregates were used to prepare normal asphalt mixtures with a nominal maximum aggregate size of 13.2 mm. Fine aggregate (2.735 g/cm^3^) and the mineral filler (2.712 g/cm^3^) were limestone.

### 2.2. Core-Shell Deicer Preparation

**Figure 2:**
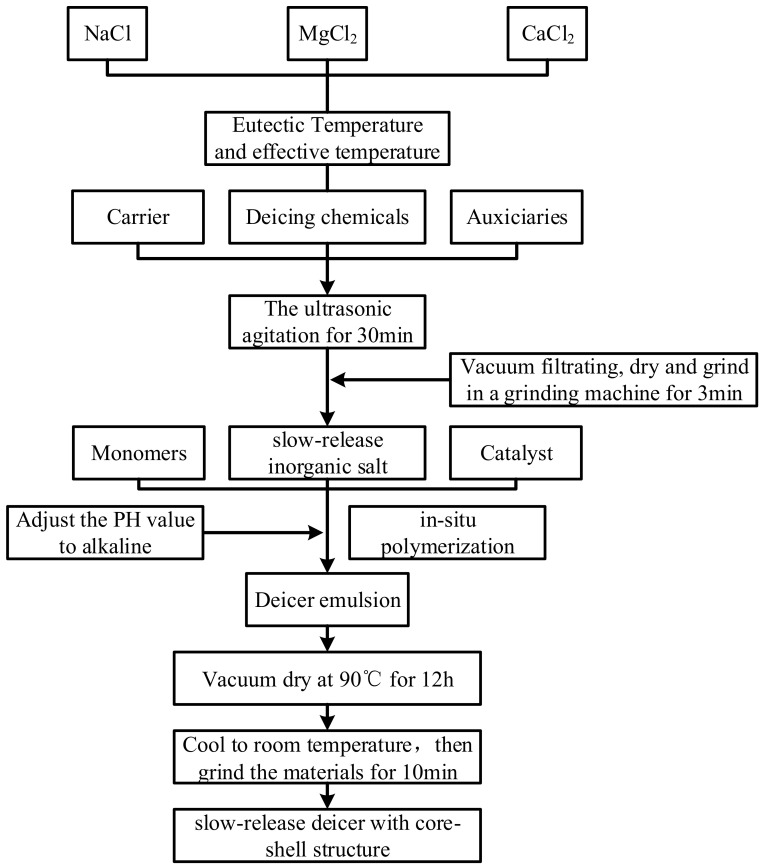
The flow chart for preparing slow-release deicer with core-shell structure.

The flow chart for preparing slow-release deicer with core-shell structure is shown in Figure 2. The preparation of slow-release deicer with core-shell structure includes two steps.

Firstly, the slow-release inorganic salt was prepared by the physical adsorption method. Saturated sodium chloride solution and diatomite nano-SiO_2_ suspension were prepared individually for use. Saturated sodium chloride solution was prepared with distilled water and sodium chloride, and the mass ratio was 100:37. Diatomite nano-SiO_2_ suspension was prepared by Nano-silica, OP-10 Nonionic Surfactant, diatomite, and ethanol, and the mass ratio was 1:4:8:160. Then, under the ultrasonic agitation, the saturated sodium chloride solution was slowly dropped into the diatomite nano-SiO_2_ suspension. The solubility of sodium chloride in water and ethanol was different, resulting in sodium chloride recrystallization. After ultrasonic agitation for another 30 min, the supersaturation sodium chloride was subjected to vacuum filtration. The final product after vacuum filtration was dried at the temperature of 120 °C and ground to obtain the fine inorganic salt powder.

Then, the core-shell structure was prepared to reduce the risks of corrosiveness and water pollution from chlorine salts. Under the action of the initiator, monomers form the shell structure on the inorganic salt powder through in-situ polymerization. The shell of polyacrylate prevented sodium chloride from contacting other materials and water.

In the in-situ polymerization, AIBN was used as the catalyst, while acrylic acid, methyl acrylate, butyl acrylate, and styrene were used as monomers. The silane coupling agent was used to modify the surface of slow-release inorganic salt. Isopropanol is a solvent that cannot dissolve slow-release inorganic salt. Isopropanol and slow-release inorganic salt could be mixed to form the solution. Triethylamine was used to adjust the PH value of the slow-release deicer.

The slow-release inorganic salt and isopropanol solvent were mixed evenly in the flask and stirred in an oil bath at 120 °C. Then, four kinds of polymer monomers, silane coupling agents, and AIBN were mixed in proportion and added to the flask through a constant pressure dropping funnel. After 3 h of reaction, the reaction temperature was reduced to 50 °C, and an appropriate amount of distilled water was added to the flask for a 1 h reaction. Finally, a small quantity of triethylamine was added to adjust the pH value of the polymer slurry to weakly alkaline. The slurry was dried in a vacuum oven at 90 °C to obtain the slow-release deicer with a core-shell structure.

### 2.3. Deicing Asphalt Mastics Preparation

SBS modified asphalt binder and core-shell deicers were used as the base materials to prepare deicing asphalt mastic. A known quantity of the asphalt binder was added to the container and heated to 180 °C, followed by the addition of the deicers in the hot asphalt over 10 min at 800 rpm with high shear mixer. In the end, the mixture was continuously stirred for 30 min using the mechanical blender.

### 2.4. Asphalt Mixture with Deicers Preparation

The aggregate gradation of the asphalt mixture is shown in Table 4. The optimal asphalt-aggregate ratio of the ordinary asphalt mixtures was 4.7%, according to the Marshall test in ASTM D6926–16 [38]. According to the volume indexes measured by different deicer replacement methods, Xia [32] found that the volume index of asphalt mixture prepared by the equal volume alternative method is almost the same as that of the ordinary asphalt mixture. Therefore, the asphalt mixture specimens incorporating 0%, 25%, 50%, 75%, and 100% deicers by volume as mineral filler were prepared. The specific replacement of 1 kg aggregate is shown in Table 5.

### 2.5. Characterization

#### 2.5.1. SEM-EDS Characterization

The microstructural characterization mainly focused on the differences between slow-release inorganic salt and slow-release deicer. A scanning electron microscope (SEM) was used to determine the morphology and microstructure of the slow-release inorganic salt and slow-release deicer. An energy dispersive X-ray spectroscopy (EDS) was used to analyze the type of elements of the deicer. The X-ray spectrum consisted of a set of peaks that represent the element type and relative content.

#### 2.5.2. DSC Characterization

The characteristic temperature at which the transition occurs is called the glass transition temperature (Tg). The glass transition temperature indirectly affects the working temperature of the slow-release deicer with a polyacrylate shell.

To determine the breaking temperature of the polyacrylate shell, the transition temperature between the glass state and the high elastic state of the polymer was measured by a differential scanning calorimetry (DSC), i.e., NETZSCH DSC 214 manufactured by the NETZSCH Group, Germany. The DSC experiments were conducted with approximately 5–10 mg of the slow-release deicer with a polyacrylate shell placed in an aluminum pan with a perforated cover. An additional empty pan was used as a reference. The slow-release deicer samples were scanned at a speed of 10 °C/min and in the temperature range of −50–50 °C. The difference in heat output was recorded to construct the DSC curve.

#### 2.5.3. TG Characterization

The thermodynamic analysis was used to investigate the thermal stability of the slow-release deicer by measuring the relationship between the mass and temperature. The decomposition temperature of the deicer was determined based on the mass loss from TG curves. TG curves were measured by thermal analysis using A NETZSCH STA 449 C. N_2_ and O_2_ were used as purge gas individually, and both flow rates were 50 mL/min. The slow-release deicer samples were heated from 50 °C to 250 °C with a heating rate of 10 °C/min.

#### 2.5.4. FTIR Characterization

In this study, the FTIR infrared spectroscopy of the asphalt, core-shell deicer, and deicer-asphalt mastic was measured by Fourier transform infrared spectrometry (FTIR) to observe the changes in the molecular structure of asphalt and explore the interactions between the asphalt and deicer. The infrared spectra were collected in the range of 400~4000 cm^−1^ with a resolution of 2 cm^−1^.

### 2.6. Performance Evaluation

#### 2.6.1. Conductivity Test

**Figure 3:**
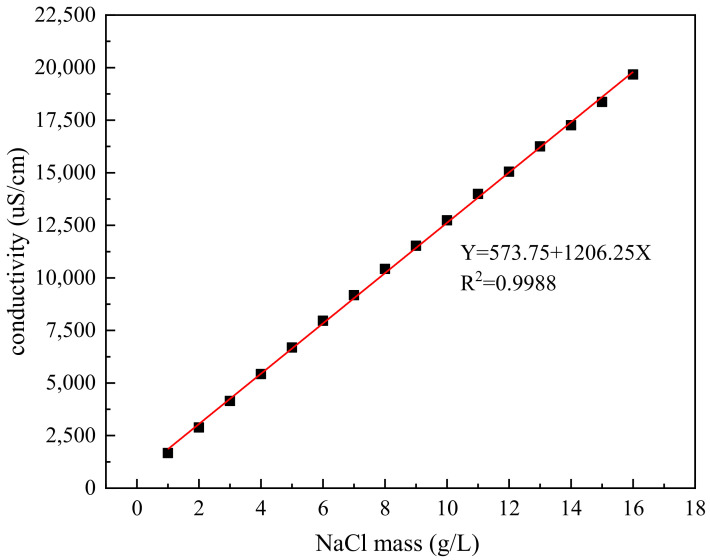
The relationship between NaCl mass concentration and conductivity.

Conductivity could reflect the concentration of ions dissolved in water. Because NaCl is a strong electrolyte and would be completely ionized in water, leading to changes in conductivity. The more dissolved salt, the higher the conductivity of the solution. The conductivity is easily affected by temperature [31]. The conductivity meter used in this study was from Shanghai Lei Magnetic (Model DDS-11A) with a temperature compensation function. When the temperature compensation regulator was adjusted to 25 °C, the measurement result was not affected by the temperature of the solution. Meanwhile, it was also equipped with a platinum black electrode (electrode constant 0.998). According to the conductivity calibration tests of the standard sodium chloride solution prepared at different concentrations, the conductivity could be replaced by the equivalent sodium chloride to reflect the release amount directly [32,33]. In the study, NaCl solution was prepared first. Then, conductivity was tested, and the relationship between NaCl mass concentration and conductivity is drawn in Figure 3, showing a high linear correlation. The fitting equation is shown as follows:

(1) $$Y=573.75+1206.25x,\;R^{2}=0.9988$$

Based on the conductivity of the slow-release inorganic salt and the deicer, the salt loaded rate and the coated rate are introduced to characterize and evaluate the effective NaCl content and the uncoated NaCl content in the deicer, respectively. Once the conductivity was measured, the NaCl mass concentration of the slow-release inorganic salt could be calculated according to Equation (1). The mass concentration of the sample could be calculated according to the mass of the samples added into the water. The loading salt rate was the ratio of NaCl mass concentration to the sample mass concentration, as shown in Equation (2).

(2) $$\sigma =\frac{\rho_{NaCl}}{\rho_{xc}}\times 100\%$$

where $\rho_{NaCl}$ and $\rho_{xc}$ are the mass concentration (g/L) of NaCl and the sample, respectively. σ is the salt loaded rate (%).

For the slow-release inorganic salt, the saturated sodium chloride solution was recrystallized on the surface and in the pores of the carrier through physical and mechanical action. The slow-release inorganic salt absorbing the water and vapor, NaCl is ionized into Na^+^ and Cl^−^ immediately. Compared with the slow-release inorganic salt, the polyacrylate shell on the surface of the deicer can effectively prevent the water and vapor from NaCl ionizing. Therefore, the difference in conductivity between the slow-release inorganic salt and the deicer could be understood to reflect the unionized NaCl which means the NaCl was covered by a shell. To estimate the efficiency of the shell, covered efficiency was defined as follows,

(3) $$\delta =\frac{EC_{0}-EC_{1}}{EC_{0}}$$

where *EC*_0_ and *EC*_1_ are the conductivity of the slow-released inorganic salt and the deicer, respectively, and δ is the coverage rate (%).

In addition, the conductivity is tested by 0.5 g material dissolved into 50 g distilled water, and the mass concentration of the material is 10 g/L.

#### 2.6.2. Moisture Absorption Rate Test

In this study, three parallel samples of 0.5 g were placed in a petri dish with a humidity of 95%, and the mass of the samples was weighed after 2 h. The moisture absorption rate was calculated according to the ratio of the mass change to the initial mass. The defined equation is shown as follows.

(4) $$\omega =\frac{m_{1}-m_{0}}{m_{0}}\times 100\%$$

Among them: ω is moisture absorption rate (%); *m*_0_ is the initial sample mass (g); and *m*_1_ is the sample mass (g) after 2 h in the humid environment.

#### 2.6.3. Ice- and Snow-Melting Performance Evaluation

##### Deicer

In this study, the tested samples included slow-release inorganic salt, slow-release deicer, and slow-release deicer with broken polymer shells. The slow-release deicer was stored in a refrigerator at −10 °C for 24 h to keep the polyacrylate shell in a glassy state. The slow-release deicer was pretreated at −10 °C and 0.7 MPa wheel load, to break the polymer shell. The wheel load cycles were set to 20 cycles, 40 cycles, and 60 cycles, to achieve the different extent of polyacrylate shell broking. In this study, 0.5 g samples were put on the surface of 50 g ice at 0 °C, and the mass of ice melting was recorded at intervals of 1 h.

##### Deicing Asphalt Mixture

These approaches to evaluate snow or ice melting performance could be divided into two categories, including quantitative analysis and qualitative analysis. The quantitative analysis was used to measure the quality of ice melting and the qualitative analysis was mainly evaluated by visual observation.

In the test, the surrounding of the sample was covered by epoxy resin. At the same time, 1 cm deep water was poured into the sleeve and frozen at −18 °C in the refrigerator for approximately 16 h. The sample was placed in a temperature chamber at 5 °C so that the whole sample could be maintained at the test temperature. Then, the frozen sample was tested for the quality of ice-melting at 5 °C in the temperature chamber and the data recorded per 15 min. The research did not end until the ice melted completely. There are 3 parallel tests.

The qualitative analysis of snow melting performance in this research was evaluated through direct observation. The field tests were conducted at the laboratory in January 2022. Multiple times rolling over snow samples containing deicer and ordinary asphalt mixture were conducted by a car. The tests were performed using a car whose inflated tire pressure was 200 kPa. The snow samples were placed on dry pavements, with the air and pavement temperatures being close to 0 °C. The test site was a parking lot located close to the laboratory facility. The parking provided shade from the sun for a large part of the day. After a snowfall, loose dendritic snow (about 10 kg) lying near the test site was first collected and then stored in a cold room at −5 °C. Before the test, about 3 kg of the stored snow was transferred to the test site and covered the samples. The tracks were visually inspected and photographed for 12 vehicle passes.

#### 2.6.4. Road Performance

Following the standard protocols by the test specification of China, the road performance of the deicing asphalt mixture in this study including the high-temperature rutting stability by the wheel tracking test, the thermal cracking resistance by the three-point bending beam test, and the moisture susceptibility by the splitting test [39].

The wheel tracking test was conducted to evaluate the high-temperature performance of asphalt mixtures. In this test, the specimens were placed on the rutting tester and were tested under a 0.7 MPa repeating wheel running for 1 h, the temperature was 60 °C.

The bending beam test was conducted to evaluate the low-temperature performance of asphalt mixtures. In the three-point bending beam test, the failure strain causing the fracture of bending beam at −10 °C is measured, which is used to indicate the thermal cracking resistance of asphalt mixtures.

The freeze-thaw splitting test was conducted to evaluate the moisture stability of asphalt mixtures. The ratio of the splitting strength after specific immersion conditioning to that of dry specimens at 25 °C is used to indicate the moisture susceptibility of the asphalt mixture.

## 3. Results and Discussion

### 3.1. Core-Shell Deicer

#### 3.1.1. SEM-EDS Characterization

**Figure 4:**
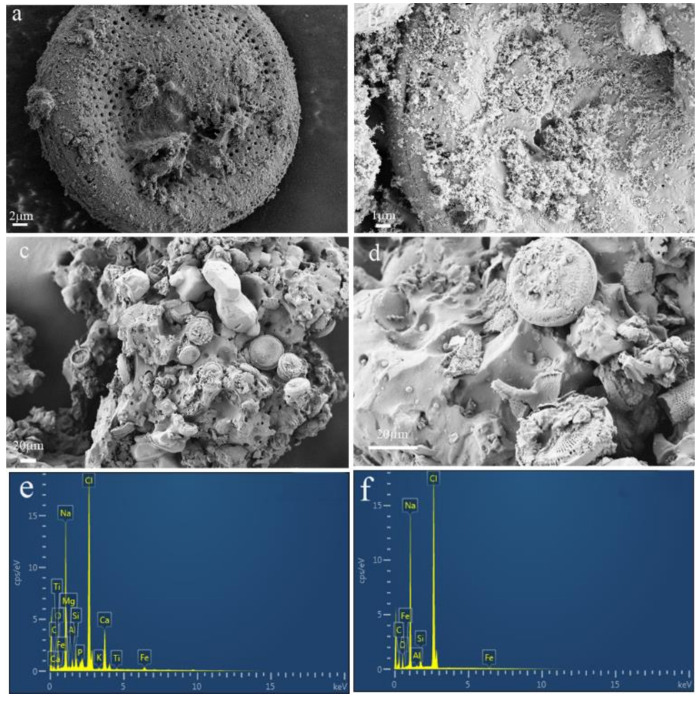
SEM/EDS of samples: (**a**,**b**,**e**): slow-release inorganic salt; (**c**,**d**,**f**): slow-release deicer.

As shown in the SEM images of slow-release inorganic salt (Figure 4a,b), it can be seen that the diatomite is circular and has a lot of small pores on the surface. The pore diameter of diatomite is in the range of 50~300 nm. A significant amount of NaCl particles after recrystallization are observed on the surface and in the pores of inorganic salt. The diatomite pore structure provides particularly good areas for sodium chloride particles. This shows that diatomite in the slow-release deicer is still circular. Through the in-situ polymerization, a shell is formed on the surface of the slow-release inorganic salt, as shown in the SEM images (Figure 4c,d). The shell prevents salt from being exposed to the air.

The SEM combined with the EDS technique is an effective tool to analyze the elemental composition of the sample. The element composition results obtained by SEM/EDS analysis, indicate there are mainly 12 elements that exist in the slow-release inorganic salt, as shown in Figure 4. The most abundant elements (>10% by quantity) include Cl, Na, Ca, C, and O, while less abundant elements (<5% by quantity) include Si, Fe, Al, Mg, Ti, P, and K. According to Table 6, the major elements (Cl, Na, Ca, C, and O) possess about 93.7% by quality, while other minor elements (Si, Fe, Al, Mg, Ti, P, and K) possess only about 6.3%. Specifically, Cl, C, and O are the most abundant elements with 29.7%, 18.5%, and 18.4% by quality, and the corresponding mass errors are 0.36%, 0.9%, and 0.23%, respectively.

It was also indicated that there are seven major elements in the slow-release deicer. The most abundant elements include Cl, Na, and C (>20% counts in the samples) and less abundant elements include O, Si, Fe, and Al (<10%). The major elements (Cl, Na, and C) possess above 90% of quality, while the minor elements (O, Si, Fe, and Al) only totally possess 10% of the whole quality. O and Si possess 9.54% by quantity among all the elements. Specifically, Cl, C, and Na possess about 38.96%, 30.86%, and 20.26% the quality, and the mass errors are 0.22%, 0.36%, and 0.12%, respectively.

The formation of a polyacrylate shell is mainly shown by the increase of carbon content and the decrease of chlorine content. By comparing the changes in carbon and chlorine content, the formation of the polymer shell can be analyzed qualitatively. In Table 6 and Table 7, comparing the carbon and chlorine contents between the slow-release inorganic salt and the slow-release deicer, it is found that the carbon content in the slow-release deicer has a more significant increase than chlorine, which indicates the polymer shell has better efficiency.

#### 3.1.2. DSC Study

**Figure 5:**
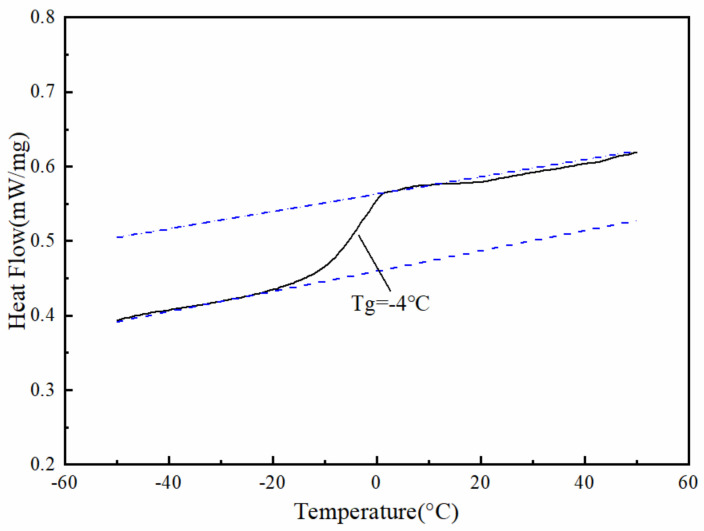
DSC curves of slow--release deicer.

The DSC testing result of the slow-release deicer is shown in Figure 3. With the variation in temperature, the state of the polyacrylate shell changes between a rubbery state and a glassy state. Based on the DSC curve, the glass transition temperature was analyzed using the coupled software. In Figure 5, there are no obvious endothermic and exothermic peaks. The starting and ending temperatures are −10.6 °C and 1.5 °C, respectively, while the glass transition temperature is −4 °C. The polyacrylate shell appeared to be in the high rubbery state when the temperature was higher than −4 °C, and in the glassy state when the temperature was lower than −4 °C. The shell of the slow-release ice-melting agent can be in a glass state if there is snow or ice on the road. Meanwhile, the polyacrylate shell breaks under the vehicle load, and the salt will be released to melt snow or deice.

#### 3.1.3. TG/DTG Analysis

Derivation of the TG curve can obtain the DTG curve of the weight loss rate with temperature changing. The TG and DTG curves of the slow-release deicer in N_2_ and O_2_ atmospheres are described in Figure 5. In the whole test temperature, there has been no significant weight loss. In the N_2_ atmosphere, when in the range of 50~212 °C, the TG curve has a weakly weightless step, and the weight loss is about 0.3%. Correspondingly, the DTG curve does not appear a significant peak. However, at around 212 °C, the weight loss start becoming obvious. In the O_2_ atmosphere, the deicer has appeared to weight loss, but the weight loss and the weight loss rate are small in the range of 50~200 °C. When the temperature is beyond 200 °C, the weight loss rate is significantly reduced.

In the range of the test temperature, the total weight loss is 0.8% and 1.4% corresponding to N_2_ and O_2_. The deicer has more obvious weight loss in O_2_ than N_2_. This may be because O_2_ provides better chemical reaction condition.

**Figure 6:**
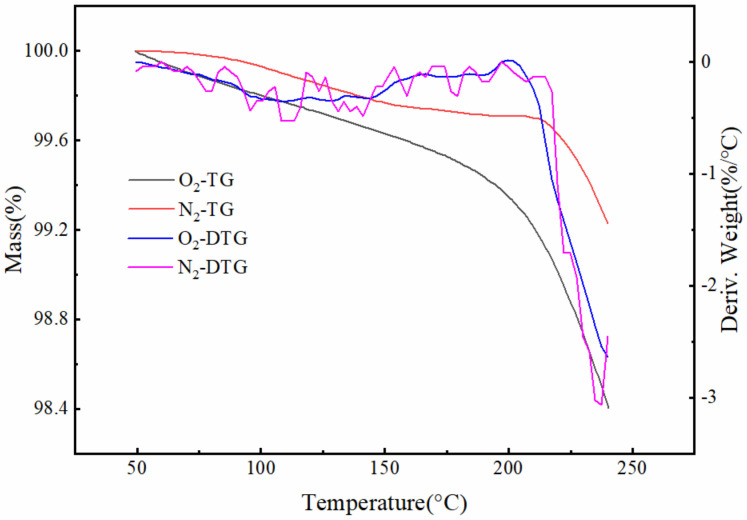
TG and DTG curves of the slow-release deicer in N_2_ and O_2_ atmospheres.

According to the DTG in Figure 6, at about 50~200 °C, the weight loss rate is small and steady. When the temperature is beyond 200 °C, the weight loss rate increases. That means that the polyacrylate shell has good thermal stability. Especially, adding the deicer into the asphalt mixture can meet the conditions of the hot mix asphalt mixture.

#### 3.1.4. Salt Loaded and Coverage Rate Study

Based on the electrical conductivity of the slow-release inorganic salt and the deicer, the effective NaCl mass concentrations of the slow-release inorganic salt and the deicers are 6.87 g/L and 0.62 g/L, respectively, according to Equation (1) and Table 8. According to Equation (2), the salt loaded rates of the slow-release inorganic salt and deicer are 68.7% and 6.2%, respectively.

For the slow-release inorganic salt, the sodium chloride solution recrystallizes into the sodium chloride crystals adsorbed on the surface and in pores of porous material. However, for the slow-release deicer with structure, the sodium chloride crystals would be covered by the shell to prevent the sodium chloride crystals from contacting water directly. In the conductivity test, the slow-release inorganic salt and the deicer respectively are mixed with the water to form a solution. The soluble salt would quickly dissolve in water and ionize into Na^+^ and Cl^−^. The conductivity of the solution increases with the increase of ions number. As for the deicer, due to the core-shell structure of polyacrylate, the sodium chloride is covered and could not dissolve in water, which affected the number of ions in the deicer. Only uncovered sodium chloride crystals could quickly dissolve in water and ionize into Na^+^ and Cl^−^, leading to the conductivity of the solution decreasing.

According to Equation (1) and (2) and Table 8, the coverage rate of the deicer with core-shell structure calculated is 85.1%. Uncovered, 14.9% NaCl will be used in the first freezing rain or snow in winter.

Based on the conductivity of the slow-release inorganic salt and the deicer, the salt loaded rate and the coated rate are introduced to characterize and evaluate the effective NaCl content and the uncoated NaCl content in the deicer, respectively. According to the evaluation index of the salt loaded rate, the NaCl mass concentration in the slow-release inorganic salt is about ten times that of the uncoated NaCl in the deicer. According to the evaluation index of the coated rate, the NaCl mass concentration in the slow-release inorganic salt is approximately six times that of the uncoated NaCl in the deicer. The main reason for the difference is that reference mass concentration is various. When it comes to the salt-loaded rate, the reference mass concertation is the concentration of the solution. According to the coated rate, the NaCl mass concertation in the slow-release inorganic salt is regarded as the reference. Therefore, by calculating the salt-loaded rate of the deicer, the NaCl mass concentration is larger than the actual, resulting in a larger NaCl mass concentration ratio calculated by the salt-loaded rate.

#### 3.1.5. Moisture Absorption Rate Analysis

In Table 9, the quality increased from 0.50 g to 0.71 g during 2 h in the humid environment. According to Equation (4), the calculated moisture absorption rate is 42.7%. The moisture absorption rate is relatively high because humidity is the main factor that affects hygroscopicity. During the experiment, the humidity of the environment is high. The vapor can penetrate deeply the deicer through the polyacrylate shell. The NaCl covered by a shell could absorb the water vapor in the deicer dissolving to form a salt solution. Meanwhile, the deicer can achieve anti-icing efficiency.

#### 3.1.6. Ice Melting Performance Evaluation

**Figure 7:**
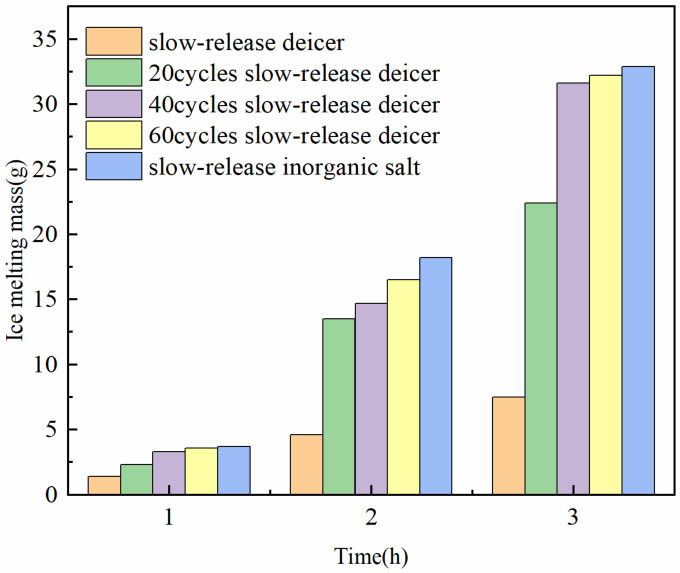
The relationship between the mass of ice-melting and time.

By measuring the mass of ice melting hourly, it can be seen from Figure 7 that the mass of ice melting off the slow-release inorganic salt is larger than others, indicating that the ice melting capacity is the best. The ice melting performance of the slow-release deicer is the worst. The effect of the ice melting of the slow-release deicer under different vehicle load periods is between the slow-release inorganic salt and the slow-release deicer. With the increase in load period, the ice melting mass and the ice melting capacity increase.

Sodium chloride salt is a strong electrolyte that is easily dissolved in water. At the temperature of 0 °C, ice starts to absorb heat from the air and partially melts into water. The salt in the slow-release inorganic salt distributed on the surface and pores of the diatomite can absorb water and dissolve to form a salt solution. According to the colligative properties of a dilute solution, the freezing point of the salt solution is lower than 0 °C, and ice continues to melt into water. The least ice melting off the slow-release snowmelt deicer is mainly contributed to the covered shell of polyacrylate polymer on the surface of the slow-release inorganic salt. The polyacrylate shell prevents the contact between salt and ice. When ice absorbs heat from air melting naturally, salt cannot dissolve to form a salt solution and reduce the freezing point. Therefore, the slow-release deicer only melts ice by the heat from the air.

If the slow-release deicer is pre-treated at −10 °C, the polyacrylate shell becomes brittle and is easily broken under the wheel load, leading to salt being exposed to the air. The loading period has a significant effect on the broken efficiency of the polyacrylate shell. With the increase of load period, the shell breaking becomes more and more obvious, and the salt in the slow-release deicer is more exposed to ice. The chance of contact between salt and water increase, and the ice mass melting capacity increases.

### 3.2. FTIR Study of Deicing Asphalt Mastics

**Figure 8:**
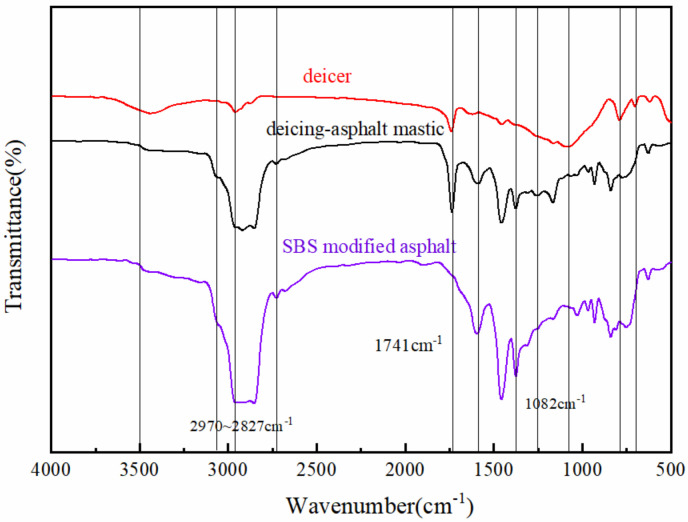
The FTIR of SBS modified asphalt, deicer, and deicer-asphalt mastics.

The modified asphalt, deicer, and deicer-asphalt mastics samples were tested by FTIR to analyze the action mechanism of sea salt erosion on asphalt, the results are shown in Figure 8. The peak at 3500 cm^−1^ in the deicer may be the O-H vibration of the hydroxyl group, which is caused by the moisture in the sample. After searching and marking peaks in the samples, the above spectra were divided into 4000~2000 cm^−1^ and 2000~500 cm^−1^ intervals. The symmetric/asymmetric stretching vibrations of C-H in the methylene group of the broad-domain peak appeared in the range of 2970~2827 cm^−1^. In the wavenumber of 2000~500 cm^−1^, the deicer and deicer-asphalt mastics have obvious carbonyl C=O characteristic peaks at 1741 cm^−1.^ The characteristic absorption peaks are the C=C stretching vibration of fused-ring aromatic hydrocarbons and the shear vibration of methylene-CH_2_. The peaks are stronger in SBS asphalt and the weakest in deicing agents. In the fingerprint area, the deicer-asphalt mastic and asphalt appear the characteristic peak of SO_2_ symmetric tensile vibration and the stretching vibration of sulfoxide-based S=O at 1163 cm^−1^ and 1031 cm^−1^, respectively. The peak is stronger than that of the modified asphalt. The characteristic absorption peaks of polybutadiene, benzene ring stretching vibration characteristic peaks, and styrene characteristic peaks appear at 926 cm^−1^, 831 cm^−1^, and 625 cm^−1^. The deicers have an obvious stretching vibration of -COO- at 1082 cm^−1^, and an obvious characteristic peak of monosubstituted benzene at 790 cm^−1^.

According to the analysis of the infrared spectra of deicer-asphalt mastics, deicer, and modified asphalt, the characteristic peaks in deicer/asphalt mastic are the superposition of characteristic peaks in deicer and SBS, and there are no other characteristic peaks. However, the strength of peaks appears different. In conclusion, the physical and chemical reactions of the deicer and SBS occurs.

### 3.3. Deicing Asphalt Mixture

#### 3.3.1. Rutting Resistance

**Figure 9:**
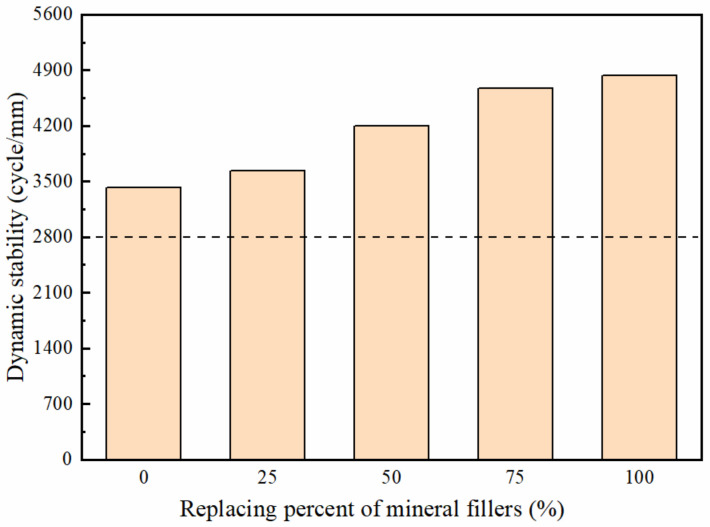
The results of wheel tracking test from asphalt mixtures with different content of deicers.

In Figure 9, the dashed lines represent the performance threshold requirements based on the Chinese specification (Ministry of Transport of the People’s Republic of China 2004). The results show that with the percentage increase of mineral filler replaced by the deicer with core-shell structure, the dynamic stability index increase. This indicates that the high-temperature stability improved as the content of the deicer increased in the deicing asphalt mixture.

Especially when the temperature is higher than the glass transition temperature, the polyacrylate shell belongs to a high elastic state and has good flexibility and ductility. According to the analysis of the TG curve, the glass transition temperature is about −3.9 °C, the wheel tracking test was conducted in a 60 °C environment, and the polyacrylate shell exhibits elasticity and ductility to resist partial deformation. In the process of testing, when the wheel load disappears, the elastic deformation will immediately recover. The total deformation is smaller, and the dynamic stability is bigger. Thus, the addition of deicer with core-shell structure causes positive effects on the high-temperature stability of the deicing asphalt mixture in general.

#### 3.3.2. Low-Temperature Performance

**Figure 10:**
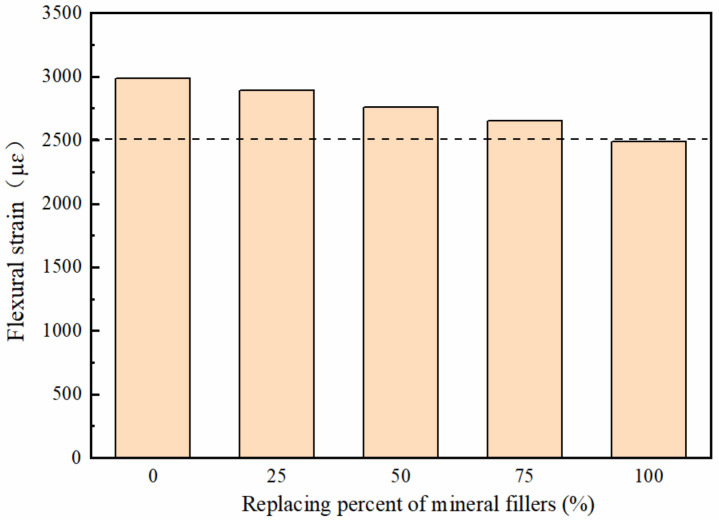
The results of the bending beam test from asphalt mixtures with different content of deicers.

The results of the low-temperature test of five replacement ratio mixtures are shown in Figure 10. It is observed that as the replacement rate of the deicer increased, the maximum flexural strain of the deicing asphalt mixture gradually decreased. In most cases, a larger flexural strain means a better low-temperature deformation. As is known, the limestone mineral filler has a better affinity with asphalt rather than the deicer. The deicer filler weakens the bond between asphalt and aggregate and thus decreases the bond strength, leading to the reduction of the low temperature cracking resistance of the deicing asphalt mixture. Besides, the deicer filler with a polyacrylate shell has better temperature-sensitivity than limestone filler, and when the environment temperature changes, the polyacrylate shell shows more obvious expansion and contraction than limestone filler. Due to the different responses to temperature change between asphalt-limestone and asphalt-deicer, the pavement produces some cracks, and the low-temperature stability decreases.

#### 3.3.3. Moisture Damage Resistance

**Figure 11:**
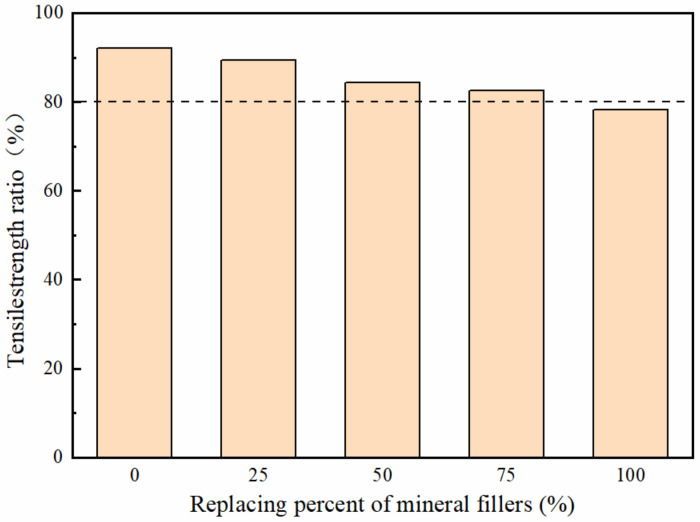
The results of the splitting test from asphalt mixtures with different content of deicers.

The results of the freeze-thaw splitting test are displayed in Figure 11. The splitting strength ratio of deicing asphalt mixtures decreased with the increase in the replacement rate. It could be found that when the replacement rate of the mineral fillers exceeds 25%, the TSR dropped significantly. Furthermore, it is shown that when the replacement rate of mineral filler by the deicer with core-shell structure reaches 100%, it is difficult to meet the performance requirements of the asphalt mixture based on Chinese requirements.

This phenomenon may be due to the fact that the surface of the deicer core-shell structure is covered with polyacrylate, which harms the adhesion with asphalt, so it decreases the adhesion between asphalt and aggregate, and finally degraded the water stability. According to the analysis of FTIR, there is no obvious chemical reaction between the polyacrylate shell and the asphalt. The reaction between deicer and asphalt in the asphalt mixture is mainly physical reactions and selective adsorption. That leads to the adhesion between asphalt and aggregate decreasing. The addition of soluble deicing chemicals in the deicing asphalt may produce some new voids and increase the volume of voids in the mixture after soluble chemicals were released, which may cause the decline of water stability. The deicer has good hydrophobicity and hygroscopicity, resulting in easy absorption of the water vapor in the air to the interface between the asphalt and the deicer. Because the water and vapor are more easily wet aggregate than asphalt, the water or vapor will compete with asphalt for the surface of aggregate, and the asphalt more easily peels off aggregate. However, each replacement rate can meet the requirements of specifications, especially when the replacement rate is less than 75%.

#### 3.3.4. Snow or Ice Melting Capacity

**Figure 12:**
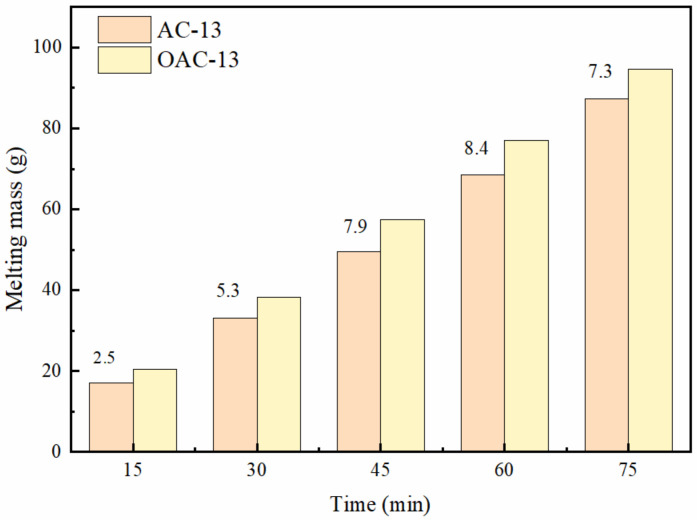
Ice melting test results of asphalt mixtures with different content of deicers.

The ice melting capacity is mainly determined by ice melting rate and ice melting mass. Figure 12 shows the mass of melted ice recorded at different times according to the ice melting test of asphalt mixture no and with deicer at 5 °C. The results show that the mass of ice melting increases gradually in the 75 min and the ice melted mass in asphalt mixture with deicer is higher than the ordinary. The ice melted mass is different in the same period. At all times, the ice melting mass is 2.5 g, 5.3 g, 7.9 g, 8.4 g, and 7.3 g higher in asphalt mixture with deicer than the ordinary, respectively. The results show that the ice melted in deicing-asphalt mixture increases firstly and then decreases. This may be related to the concentration of deicing salt, the molecular weight of the ionizable salt, and the influence of the ions on ice crystallization. In the first 15 min, the ice only contacted the Marshall samples to adjust itself to the temperature at 5 °C, and the salt concentration is low. In the process, the ice melting is determined by the surrounding temperature. With the melted ice quality increasing, more and more water penetrated the sample to dissolve the salt, improving the aqueous solution concentration. At the same time, the deicing salt in water ionizes to form Cl^−^ and Na^+^, destroying the ice structure and leading the ice to melt, because the molecular weight of deicing salt is small and its solubility is large. According to the relationship between ion diameter and diffusion rate, the ion diameter is small, and the diffusion rate is large. Thus, considering the osmotic pressure and Fick’s law, the ion diffuses from high concentration to low concentration decreasing the ion concentration in the aqueous solution, leading to the ice melting capacity decline.

**Figure 13:**
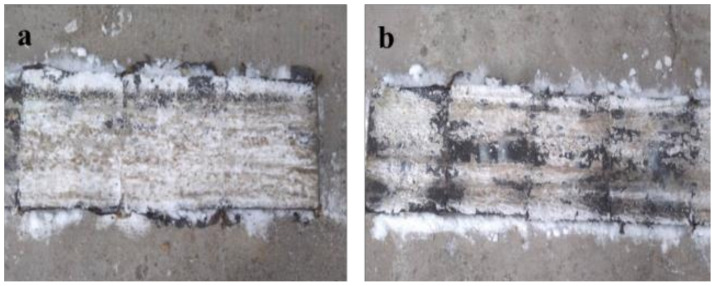
Field trials ((**a**): the ordinary asphalt mixture; (**b**): the deicing asphalt mixture).

As Figure 13 shown, with the addition of deicer, salted snow was soft and more easily detached from the pavement surface. Compacted dry snow was very hard in the ordinary asphalt mixture, and as a result, it was barely easily removed from the pavement. Snow in the deicing asphalt mixture was weaker than the dry compacted snow. Under the action of vehicle loads, snow in the deicing asphalt mixture was slushy and extremely weak. Moreover, after 12 passes by the test vehicle, the snow had already been melted and squeezed out.

## 4. Conclusions

A novel core-shell deicer with a slow-release priority is prepared to reduce the impact of inorganic salt on the surroundings. Based on the results obtained in this study, the following conclusions can be drawn:

(1) The slow-releasing deicers with core-shell were prepared through in-situ polymerization, which have good thermal stability and ice-melting performance and can meet the requirements in winter and effectively reduce the loss of salts in rain and humidity.
(2) The rutting susceptibility got better, while the low temperature cracking resistance and the water stability of mixtures got worse, with the increasing replacement of deicer filler. Considering the road performance, the replacement rate of the deicer is less than 75%.
(3) According to the results of the ice melting test and field verification, the deicing asphalt mixtures would obtain a remarkable improvement in ice melting capacity and slow the bonding strength between mixture and pavement.
(4) Of course, in this study, there are some limitations. Only sodium chloride is considered, while other chlorine salts are not considered from the perspective of deicer material properties. Research on the durability of deicer and deicing asphalt mixture and evaluation of the cost–benefit ration have not been carried out, which will be conducted in the future.

## 5. Patents

Guo P., Feng Y.X., et al. Polymer shell coating material, polymer coating slow-release inorganic salt and preparation methods thereof [P], CN: 201811057835, 2018.

## Figures and Tables

**Table 1:** The eutectic and effective temperature of chloride salt [37].

Chemical Name	Eutectic Temp. (°C)	Effective Temp. (°C)
sodium chloride (NaCl)	−21	−9
magnesium chloride (MgCl_2_)	−33	−15
calcium chloride (CaCl_2_)	−51	−29

**Table 2:** Basic properties of SBS modified asphalt.

Test Items	25 °C Penetration (0.1 mm)	5 °C Ductility (cm)	Softening Point (°C)	Penetration Index
Test values	68.5	37	65	0.6

**Table 3:** Basic properties of aggregates and mineral fillers.

Property	Test Values
Crushed value (%)	16.3
Los Angeles abrasion loss (%)	13.5
Soundness (%)	4.0
Flat and elongated particles (%)	4.2
Apparent specific gravity	2.712 (limestone)
	2.386 (deicer)

**Table 4:** Aggregate gradation used in this study.

Passing Percent	Sieving Size (mm)									
	16	13.2	9.5	4.75	2.36	1.18	0.6	0.3	0.15	0.075
Upper limit gradation (%)	100	100	85	68	50	38	28	20	15	8
Design gradation (%)	100	96	70.3	42.5	30.5	22.0	17.0	11.5	8.5	6
Lower graduation (%)	100	90	65	38	24	15	10	7	5	4

**Table 5:** The mass of deicer and mineral in aggregate of 1 kg by equal replacement.

Deicer Volume	0%	25%	50%	75%	100%
Mineral (g)	60	45	30	15	0
Deicer (g)	0	13.2	26.4	39.6	52

**Table 6:** EDS quantitative results of the slow-release inorganic salt.

Elements K Line	C	O	Na	Si	Cl	K	Al	Mg	Ca	Ti	Fe	P
wt%	18.51	18.42	17.16	2.17	29.3	0.29	0.99	0.21	10.28	0.32	2.32	0.03
wt% Sigma	0.9	0.34	0.23	0.06	0.36	0.05	0.05	0.04	0.16	0.07	0.16	0.05

**Table 7:** EDS quantitative results of the slow-release deicer.

Elements K Line	C	O	Na	Al	Si	Cl	Fe
wt%	30.86	7.16	20.26	0.15	2.38	38.96	0.22
wt% Sigma	0.36	0.10	0.12	0.02	0.03	0.22	0.06

**Table 8:** Test results of solution conductivity.

Category	Core Material			Deicer		
Value of test (µS/cm)	8960	8720	8890	1340	1290	1320
Average value (µS/cm)	8857			1317		

**Table 9:** Data recording and analysis of moisture absorption experiment.

	Moisture Absorption Before (g)			Moisture Absorption After (g)		
Average (g)	0.5002	0.4974	0.5012	0.7134	0.7118	0.7141
	0.4996			0.7131		
ω (%)	42.7					

## Data Availability

Not applicable.
